# Solvent Effects on Radical Copolymerization Kinetics of 2-Hydroxyethyl Methacrylate and Butyl Methacrylate

**DOI:** 10.3390/polym11030487

**Published:** 2019-03-13

**Authors:** Loretta A. Idowu, Robin A. Hutchinson

**Affiliations:** Department of Chemical Engineering, Queen’s University, 19 Division St., Kingston, ON K7L 3N6, Canada; 16lai@queensu.ca

**Keywords:** radical polymerization, polymerization kinetics, copolymerization, reactivity ratios, PLP-SEC, radical propagation, 2-hydroxyethyl methacrylate, hydrogen bonding, solvent effects.

## Abstract

2-Hydroxyethyl methacrylate (HEMA) is an important component of many acrylic resins used in coatings formulations, as the functionality ensures that the chains participate in the cross-linking reactions required to form the final product. Hence, the knowledge of their radical copolymerization kinetic coefficients is vital for both process and recipe improvements. The pulsed laser polymerization (PLP) technique is paired with size exclusion chromatography (SEC) and nuclear magnetic resonance (NMR) to provide kinetic coefficients for the copolymerization of HEMA with butyl methacrylate (BMA) in various solvents. The choice of solvent has a significant impact on both copolymer composition and on the composition-averaged propagation rate coefficient (k_p,cop_). Compared to the bulk system, both *n*-butanol and dimethylformamide reduce the relative reactivity of HEMA during copolymerization, while xylene as a solvent enhances HEMA reactivity. The magnitude of the solvent effect varies with monomer concentration, as shown by a systematic study of monomer/solvent mixtures containing 50 vol%, 20 vol%, and 10 vol% monomer. The observed behavior is related to the influence of hydrogen bonding on monomer reactivity, with the experimental results fit using the terminal model of radical copolymerization to provide estimates of reactivity ratios and k_p,HEMA_.

## 1. Introduction

Acrylic copolymers are commonly used as binder resins in automotive coatings because of their contributions to film properties, including adhesion, strength (crack resistance), appearance, and chemical and water resistances [1]. These resins are prepared through the free radical polymerization (FRP) of a mixture of acrylate, methacrylate, and other vinyl unsaturated compounds. Led by an expectation from the consumer for improvement in coatings characteristics balanced with the burden of rising raw material cost, the industry is constantly seeking to improve their recipes and methodologies. In the pursuit of these goals, solvent-borne coatings employ monomers with reactive functionalities, in order to reduce the amount of solvent required in the formulation by also reducing the average molecular weight (MW) of the polymer being produced. Such a monomer is 2-hydroxyethyl methacrylate (HEMA); hydrophilic and easily polymerized, its primary alcohol allows for easy post modification (such as crosslinking) to impart the durability and strength needed in the final coating. In order to distribute the crosslink sites along the polymer chain, as well as to reduce the impact of its higher relative cost, the fraction of HEMA used as a comonomer in automotive coatings is kept relatively low. A knowledge of its copolymerization kinetics with other common acrylate, methacrylate, and styrenic monomers is required in order to understand how the functional groups are distributed in the acrylic resin.

FRP is used to produce these materials, owing to its lower cost, its tolerance to trace impurities, and its robustness to monomer choice. In FRP, however, the lifetime of a single chain is a fraction of a second, while the overall reaction time to completely convert the monomer to polymer is typically several hours. Thus, there is the need to tightly control reactor conditions so that the chains produced at the beginning of the batch are of similar composition and MW as those produced at the end. In other words, it is necessary to be able to predict how the relative consumption rates of monomers and initiator in the reactor vary with reaction conditions. The reaction system is further complicated by the presence of hydroxy-functional monomers, as the solvent choice affects the FRP copolymerization kinetics.

A very important parameter controlling both polymerization rate and polymer molecular weight is the propagation rate coefficient (k_p_), which can be determined by pulsed laser polymerization in conjunction with size exclusion chromatography (PLP-SEC). This specialized method developed by Olaj et al. [2] allows for an accurate determination of k_p_ from the molecular weight distribution (MWD) of the resultant polymer. A mixture of monomer and photoinitiator is exposed to a series of laser pulses at a set repetition rate, with each pulse creating a fresh population of radicals in the system. While initiation only occurs with each pulse, propagation and termination of radicals proceed continuously between the pulses. At the subsequent pulse, a fresh set of radicals is created, causing a burst of termination to occur with the previously created radicals that have survived termination for the t_0_ seconds between pulses:

(1) $$L_{i}{=\mathrm{ik}}_{p}\left[M\right]t_{0}.$$

The dead chains formed at that instant have a specific chain length (L_i_), which is the product of k_p_, monomer concentration [M], assumed to be constant for the experiment, as monomer conversion is kept low, generally < 5%), and t_0_. The isolated polymer product from the PLP experiment analyzed by SEC shows distinct features corresponding to L_i_, with the position of the inflection point of the second peak (L_2_) having a molecular weight approximately twice the value of the first (L_1_) [2,3,4,5,6,7,8,9].

Validated by extensive theoretical and experimental investigations, the PLP technique has been successfully used to determine the propagation coefficients for various monomers, with results summarized by a series of IUPAC-endorsed publications providing benchmark values for styrene [3], methacrylates [4,5,6], acrylates [7,8], and vinyl acetate [9]. With these studies and more, variations within and between the acrylate and methacrylate families of monomers are generalized. In the methacrylate family, an increase in k_p_ by ~30% is seen with an increase in the size of ester-side chain, with no significant change in the activation energy observed [5]. Similar behavior is seen for the acrylate family, although the acrylate chain-end propagation rate coefficients are roughly 50 times greater than those of methacrylates [8]. The most relevant observation to this work is the variation in trend by monomers containing an OH-group. For example, HEMA [10] and 2-hydroxethyl acrylate (HEA) [11] are characterized by k_p_ values up to a factor of two higher than the values for the other members of their respective families, a result attributed to intermolecular hydrogen-bonding between monomer units that reduces the electron density (and hence increases reactivity) of the double bond [11,12,13,14]. The PLP-SEC technique was also used to demonstrate that an H-bonding solvent such as *n*-butanol increases the k_p_ value of *n*-butyl methacrylate (BMA) by greater than 50% [12]. The influence of solvent is strongest when hydrogen bonding between solvent and monomer occurs, as solvent influences on k_p_ are limited to at most 20% in the absence of H-bonding [13].

Recent efforts have explored the influence of H-bonding on copolymerization kinetics, both rate and copolymer composition. Compared with common alkyl methacrylates such as BMA and dodecyl methacrylate (DMA) copolymerized with styrene (ST), the copolymerization of HEMA with ST in bulk is characterized by a higher incorporation of HEMA into the ST-methacrylate copolymer compared with the other systems [14]. A follow-up study determined that solvent choice greatly influences both copolymer composition and the composition-averaged propagation rate coefficient (k_p,cop_) for ST/HEMA, but has a negligible influence on the kinetics of ST/BMA copolymerization [15]. In particular, polar solvents were shown to reduce the relative reactivity of HEMA during copolymerization, but not influence BMA reactivity. The findings aligned with the hypothesis by Beuermann [12,13] that competitive monomer/monomer and monomer/solvent H-bonding impacts the electron density around the double bond, and thus its reactivity to radical addition.

While similar behavior has been observed when HEMA or 2-hydroxyethyl acrylate (HEA) are copolymerized with other acrylates and methacrylates [16,17], the influence of solvent choice on the system reactivity ratios has yet to be generalized. Rooney and Hutchinson [18] recently hypothesized that both the type and the amount of solvent significantly impact relative monomer reactivities by influencing the extent of H-bonding interactions between functionalized monomers (HEMA, HEA) and the same units incorporated into the copolymer chain. However, more data are required to determine whether this representation can be applied across a broad range of conditions.

With that goal in mind, the copolymerization of HEMA with BMA is an attractive system to study, as the relative incorporation rates of monomers in methacrylate–methacrylate copolymerization are equal in the absence of H-bonding effects, such that copolymer composition matches that of the monomer mixture. BMA is widely used for coatings owing to its lower cost and the fact that its hydrophobic nature traits make it a desirable monomer to copolymerize with HEMA. Liang et al. [19] reported that *n*-butanol (BUOH) boosted BMA incorporation such that it eased towards the same reactivity as HEMA, whereas the reactivity of HEMA was suppressed in dimethylformamide (DMF) because of the disruption of H-bonding between HEMA molecules. These results were in line with the solvent effects reported in the comparison of ST/HEMA and ST/BMA copolymerization kinetics [14,15]. However, the experimental dataset was limited and the fraction of solvent relative to the BMA/HEMA mixture was kept constant at 50 vol% [19]. In this paper, we conduct PLP-SEC experiments at two temperatures to completely determine how copolymer composition and k_p,cop_ are affected by the solvent to monomer ratio as well as the solvent type. In addition, the ability of the terminal model to represent the dataset is assessed.

## 2. Materials and Methods

The chemicals HEMA (97% purity, containing ≤250 ppm monomethyl ether hydroquinone inhibitor (MEHQ)), BMA (99% purity, containing 10 ppm MEHQ as inhibitor), BUOH (≥99.9% purity), DMF (99.8% purity), THF (tetrahydrofuran containing 250 ppm BHT as inhibitor, ≥99.0% purity), and photoinitiator DMPA (2,2-dimethoxy-2-phenylacetophenone, 99% purity) were obtained from Sigma-Aldrich and used as received. Xylene (isomeric mixture, ≥98.5% purity) procured from Fisher Scientific and DMSO-d_6_ (dimethyl sulfoxide-d_6_, 99.9% D) from Cambridge Isotope Laboratories Inc. (Tewksbury, MA USA) were all also used as received.

Polymerizations were performed to low conversions in a pulsed laser setup following previously established procedures [14,15,16,17,18]. A Coherent (Santa Clara, CA, USA) Excimer Xantos XS Laser (XeF as the reactive gas) capable of producing a 351 nm laser pulse was used as the radiation source, with 5 ns pulse duration, 500 Hz pulse repetition capability, and 6 mJ maximum energy per pulse. The laser beam was reflected into a ThorLabs Inc. (Newton, NJ, USA) Q3500 µL quartz sample cell used as the PLP reactor.

BMA/HEMA comonomer mixtures of varying composition (vol %) were prepared with 5 mmol/L DMPA photoinitiator. The comonomer mixtures were then diluted to 10% (90 vol% solvent), 20% (80 vol% solvent), and 50% (50 vol% solvent) for the study, with some experiments also conducted in bulk (0% solvent). The comonomer/solvent mixture (~2 mL) was added to the cell and exposed to the laser energy with temperature controlled by a circulating oil bath and measured in the cell to be at ±1 °C of the desired set point (50 or 80 °C). The conversions save for bulk (no solvent used) were kept below 3% to avoid composition drift. Conversions in bulk were slightly higher but below 10%, still providing reliable kinetic data [16,17,20]. The resulting polymer was dried under air to aid in the removal of supernatant solution (solvent and unreacted monomer mixture).

To determine propagation kinetics (k_p,cop_)*,* size exclusion chromatography (SEC) was used to analyze the molecular weight distribution (MWD) of the polymer. Polymer samples analyzed for propagation kinetics were dissolved in THF at a concentration of 3 mg/mL and passed through 0.2 µm filters for size exclusion chromatography (SEC) analysis. As copolymer samples with greater than 70 mol% HEMA were insoluble in THF, k_p_ values of these HEMA-rich copolymers could not be evaluated. The SEC setup consisted of a Waters Corporation (Milford, MA, USA) 2960 separation module with a Waters 410 differential refractometer (RI detector) and a Wyatt Instruments (Santa Barbara, CA, USA) Dawn EOS 690 nm multi-angle light scattering detector (LS detector). Calibration for the RI detector was performed using polystyrene standards that ranged from 890–3.55 × 10^5^ g/mol, while the MWs of the copolymers were calculated via universal calibration using a weighted average of the known Mark–Houwink parameters for poly(HEMA) and poly(BMA) homopolymer [14,19]. The LS output was determined from known dn/dc values, and thus is independent from the standards used to calibrate the RI detector. A comparison showed that the k_p,cop_ values estimated from the two detectors were within 10% of each other. As polymer concentrations were sometimes too low for reliable analysis by the LS detector, the RI results, which also showed better reproducibility, are reported in the study.

Differentiation of the resultant MWD slices gives values for the inflection points from which k_p,cop_ is calculated according to Equation (1), modified for copolymerization as follows:

(2) $$k_{p,\mathrm{cop}}=\frac{{\mathrm{MW}}_{i}}{i\phi_{\mathrm{mon}}\rho t_{0}},$$

where MW_i_ is the polymer molecular weight at the ith inflection point maximum determined from SEC analysis, ϕ_mon_ is the volume fraction of the monomer in the monomer/solvent mixture, ρ is the monomer mixture density (assuming volume additivity), and t_0_ is the time between the pulses (inverse of the laser frequency). Table 1 summarizes the necessary monomer densities, dn/dc values, and Mark–Houwink (K and a) constants required to analyze the system. On the basis of previous practices [14,15,16,17], copolymer values are estimated using a weighted average of the known homopolymer values.

**Figure 1:**
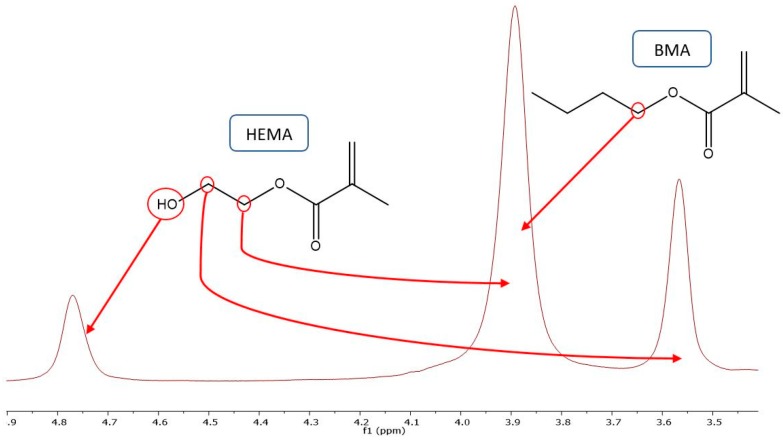
Expanded proton nuclear magnetic resonance (NMR) spectrum showing peaks used for determination of butyl methacrylate (BMA)/2-hydroxyethyl methacrylate (HEMA) copolymer composition.

Copolymer composition was determined by proton nuclear magnetic resonance (NMR). After air drying, the samples were precipitated in an appropriate solvent/non-solvent mixture. Copolymer produced in BMA/HEMA mixtures varying between 100:0 and 80:20 were precipitated in a 1:1 *v*/*v* methanol/water mixture and the remaining compositions in a diethyl ether/heptane mixture. The excess solvent was decanted and the samples dried in a vacuum oven at 60 °C for at least 24 h. The polymer was then dissolved in DMSO-d_6_ at ~10 mg/mL for proton NMR analysis conducted on a 500 MHz Bruker at room temperature. An example spectrum is shown in Figure 1, with copolymer fraction determined using an integral analysis of side chain proton signals according to the following:

(3) $$F_{\mathrm{HEMA}}=\frac{2\int {OH|}_{4.8ppm}}{\int {CH_{2}|}_{3.9ppm}}\;or\;F_{\mathrm{HEMA}}=\frac{\int {CH_{2}|}_{3.56ppm}}{\int {CH_{2}|}_{3.9ppm}}.$$

The estimates from the two calculations were in good agreement (less than 5% difference) with average values reported. Monomer presence (if any) was determined by integration of the double bond peaks (5.68 and 6.06). These peak integration values were subtracted from polymer integrations, to ensure composition was determined based on polymer content alone. Some samples had interference with the peak at 3.56 ppm owing to a water peak overlap from the DMSO-d_6_. For these cases, a 600 MHz Bruker with a water peak removal function was used and though the differences were kept under 10%, only the OH peak calculation values were reported.

## 3. Results and Discussion

### 3.1. Analysis of Copolymer Composition

Samples analyzed for copolymer composition were synthesized in the PLP setup at 50 °C, with a few samples produced at 80 °C also characterized. In accord with previous studies [16,21], copolymer composition was independent of temperature in this range. Experiments were conducted for bulk HEMA/BMA, as well as for comonomer mixtues diluted with DMF, xylenes, and BUOH to 10 vol%, 20 vol%, and 50 vol% monomer content.

Each data set is fit by the well-known Mayo–Lewis terminal model [22], which relates copolymer molar composition (F_1_) to the corresponding comonomer molar composition (f_1_) in terms of the reactivity ratios (r_1_ and r_2_) that describe the relative monomer reactivity in the system.

(4) $$F_{1}=\frac{r_{1}{\left(f_{1}\right)}^{2}+f_{1}f_{2}}{r_{1}{\left(f_{1}\right)}^{2}+2f_{1}f_{2}+r_{2}{\left(f_{2}\right)}^{2}},$$

with

(4a) $$r_{1}=\frac{k_{p,11}}{k_{p,12}}\;,\;r_{2}=\frac{k_{p,22}}{k_{p,21}}\;,\;f_{1}=\frac{\left[M_{1}\right]}{\left[M_{1}]+[M_{2}\right]},\;f_{1}+f_{2}=1,$$

and k_p,ij_ the propagation coefficient for the addition of monomer j to radical i.

**Figure 2:**
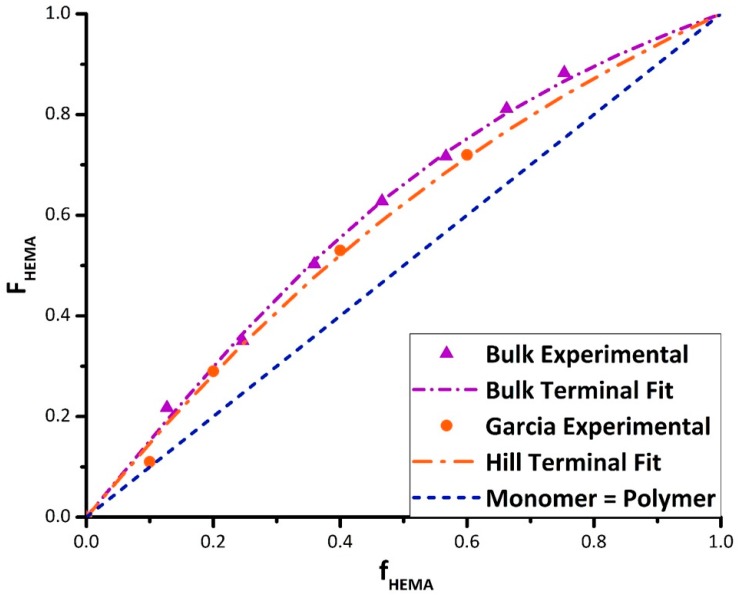
Mayo Lewis plot of HEMA molar composition in the copolymer (F_HEMA_) vs. comonomer mixture (f_HEMA_) for BMA/HEMA in bulk, compared with literature values reported by Fernández-García et al. [23] and Hill et al. [24]. Experimental data given by symbols, terminal model fits by dotted lines.

A set of experiments were run in bulk to establish a baseline relationship between comonomer and copolymer composition in the absence of solvent. The reactivity ratios were calculated using non-linear parameter estimation in Origin Lab software and confirmed with parameter estimation routines in MATLAB. The current data are in reasonable agreement with results reported by Fernández-García et al. [23] and Hill et al. [24] for bulk BMA/HEMA, as demonstrated in Figure 2. The best fit reactivity ratios (with 95% confidence intervals) from the data obtained in this study are r_HEMA_ = 2.27 ± 0.18 and r_BMA_ = 0.66 ± 0.06, whereas the values from Hill et al., based on fewer data points and reported without confidence intervals, are r_HEMA_ = 1.73 and r_BMA_ = 0.65. The fit of the combined dataset yields estimates of r_HEMA_ = 2.25 ± 0.20 and r_BMA_ = 0.72 ± 0.07, within confidence limits of the values estimated using the new data alone.

In line with previous studies, the polar HEMA has higher incorporation into the copolymer than the non-polar BMA. While this enhanced reactivity is explained by H-bonding between HEMA molecules, it is somewhat surprising, as discussed by Rooney and Hutchinson [18], that HEMA does not also enhance BMA reactivity to the same extent through H-bonding with the BMA carbonyl group; previous work has shown that BUOH as a solvent enhances BMA reactivity both during homopolymerization [12] and when copolymerized with styrene [15]. The result suggests that the intermolecular bonding between HEMA units is stronger than that between HEMA and BMA.

Recognizing the influence of H-bonding on HEMA relative reactivity, previous studies have studied the possibility of modifying this effect by introducing solvents that introduce competitive H-bonding or disrupt H-bonding in the system. This work goes further, varying not only solvent type but also monomer concentration (solvent fraction) to systematically explore the extent of H-bond influences on reactivity. The solvents used in this work—*n*-butanol (BUOH), dimethylformamide (DMF), and xylene—were chosen based on previous research that highlighted their impact on related systems [15,16,17].

**Figure 3:**
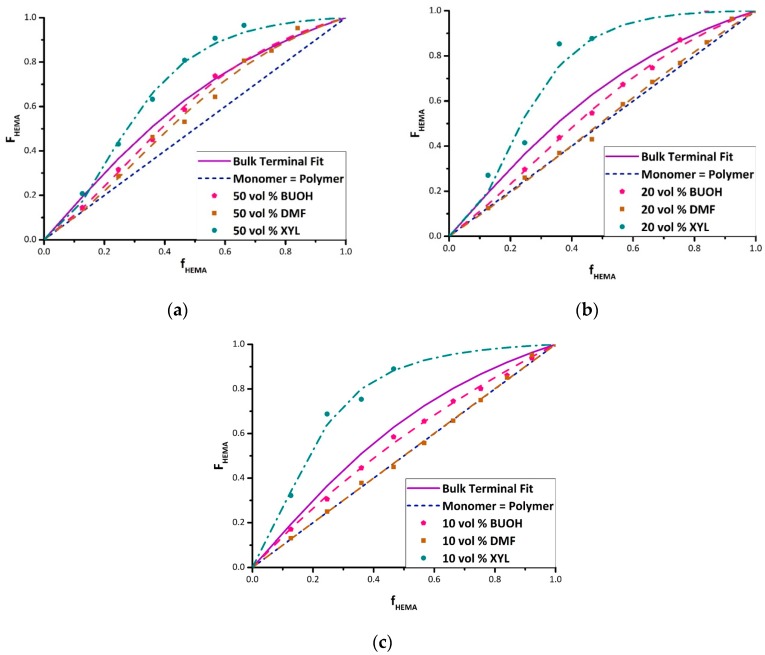
BMA/HEMA Mayo Lewis plots of HEMA molar composition in the copolymer (F_HEMA_) vs. comonomer mixture (f_HEMA_), grouped by vol% monomer in the mixture: (**a**) 50 vol% monomer in solvent; (**b**) 20 vol% monomer in solvent; and (**c**) 10 vol% monomer in solvent. Lines are the best-fit representation of experimental data points according to the terminal model using reactivity ratios summarized in Table 2. Each plot contains the curve for the bulk system (solid line), and the F_HEMA_ = f_HEMA_ diagonal (dashed line) indicating equal comonomer reactivity. BUOH—*n*-butanol; DMF—dimethylformamide; XYL—xylene.

Using the bulk copolymer composition results as a reference, experiments with BMA/HEMA in the three solvents were carried out at comonomer/solvent volume ratios of 50/50, 20/80, and 10/90. Although in some cases it was not possible to cover the complete composition range because of heterogeneity (i.e., polymer precipitation) in the system, the data were used to estimate reactivity ratios for all conditions. Figure 3 summarizes the complete set of experimental results in the form of Mayo–Lewis plots, with data tabulated as Supporting Information (Tables S1 and S2). The figure also presents the best-fit representation of the data according to the terminal model, with the corresponding estimates of reactivity ratios summarized in Table 2. The values were determined with some high HEMA data points excluded during the fit because of concerns regarding NMR integrations and the precipitation of copolymer from the solvent/monomer mixture [25].

The data shows the expected dampening effect of polar solvents BUOH and DMF on HEMA incorporation [15,19], with the extent of the variation from the bulk system dependent on the solvent level. In addition, a boosting effect of xylene on HEMA reactivity is observed. For example, with 20 vol% monomer in solution (Figure 3b) and f_HEMA_ = 0.47, the HEMA fraction incorporated into the copolymer increases from its bulk value of 0.63 to 0.88 in xylene, but decreases to 0.55 in BUOH and 0.43 in DMF. Previous studies [15,16,17] have attributed the decrease in HEMA incorporation (relative to bulk) to the role of the solvent in enhancing (BUOH) or disrupting (DMF) H-bonding, although a clear interpretation of xylene on relative reactivity has not been established. Further discussion of the data, organized by solvent, follows.

As studied by Beuermann [12,13], the OH group in BUOH undergoes hydrogen bonding with the BMA carbonyl group, decreasing the electron density of the double bond, thereby increasing its reactivity towards radical attack, as demonstrated by the increase in its homopropagation rate coefficient. The role of BUOH in the presence of HEMA is less clear, as both species can participate in H-bonding. At 50 vol% solvent, the addition of BUOH enhances the reactivity of BMA to a greater extent than HEMA does, as the fraction of HEMA incorporated into the copolymer decreases towards the diagonal compared with in bulk for f_HEMA_ < 0.5; at higher HEMA fractions, however, the copolymer composition is the same as measured for the bulk system. Increasing the BUOH fraction to 80 vol% and 90 vol% lowers the HEMA incorporation over the complete composition range, such that the best-fit curves lie entirely below that of the bulk case. The decrease in HEMA incorporation is largely captured by a decrease in the HEMA reactivity ratio (see Table 2), although HEMA monomer addition to a HEMA radical is still favored over BMA monomer addition even at a high BUOH fraction. Though the solvent contribution towards H-bonding boosts the reactivity of BMA, it is not selective and also increases the probability of increased H-bonding for HEMA, allowing the preferential incorporation of HEMA to persist.

Adding DMF as solvent also decreases the HEMA incorporation into the copolymer relative to bulk. DMF has a greater influence than BUOH, however, such that the reactivity of BMA becomes equal to that of HEMA (i.e., copolymer composition along the diagonal in Figure 3) for 20 vol% and 10 vol% monomer in solution; the corresponding reactivity ratio estimates decrease to unity within experimental error (Table 2). This result can be attributed to the aprotic nature of DMF that serves to disrupt the H-bonding between HEMA molecules rather than increasing BMA reactivity [15,19]. DMF levels the playing field for the two monomers, completely eliminating the higher relative reactivity for HEMA seen in bulk copolymerization with BMA.

As a non-polar solvent, xylene might be expected to be inert and hence yield the same copolymer composition as measured in bulk. However, there is a substantial increase in the relative reactivity of HEMA in xylene compared with the bulk case seen at all solvent levels. Schier and Hutchinson [16] and Ito et al. [26] also observed that HEMA (or HEA) has an abnormally high relative reactivity when copolymerized in aromatic compounds such as toluene and xylene that is not seen in other non-polar solvents. The reason for this behavior is not evident. Aromatic compounds are capable of forming weak hydrogen bonds owing to their π electrons, with substitution by a methyl group increasing the donor ability of the ring [27]. It has been hypothesized that this reduces the electron density of the HEMA double bond and further enhances its reactivity, as suggested in a recent computational study [28]. However, it is unlikely that this effect is stronger than that introduced by intermolecular H-bonding between HEMA units.

Others [23,29] propose that solvent effects in HEMA copolymerization result from a bootstrap effect, where the preferential solvation of HEMA next to the growing chain causes a higher localized concentration. This microphase separation isolates pockets of monomer, which leads to preferential HEMA incorporation without any change in the actual reactivity ratios. However, a careful study by Ito et al. [26] argues that the magnitude of the change is too large to be explained by the bootstrap effect alone. Their investigation of HEMA copolymerized with dodecyl methacrylate (DMA) reports very similar results to what is found in the current study for BMA/HEMA; with all experiments conducted at a total monomer concentration of 0.5 mol/L, the reactivity of HEMA is abnormally high in benzene (r_HEMA_ = 11, r_DMA_ = 0.7), HEMA is more reactive than DMA in *tert*-BUOH (r_HEMA_ = 1.6, r_DMA_ = 0.5), and copolymerization in DMF leads to equal incorporation of the two monomers (r_HEMA_ ≅ r_DMA_ ≅ 1.0). These reported reactivity ratio values for HEMA/DMA are in excellent agreement with the Table 2 best-fit values for HEMA/BMA with 10 vol% and 20 vol% monomer in solution. The study by Ito et al. [26] determined that the more than ten-fold increase in r_HEMA_ greatly surpassed the increased mean degree of HEMA aggregation in benzene, which was measured to be a factor of two based on cryoscopic measurements. Thus, it was concluded that the bootstrap effect alone cannot explain the magnitude of the increased HEMA incorporation. Rooney and Hutchinson [18] propose that the increased reactivity of HEMA results not from monomer aggregation, but rather from HEMA hydrogen bonding between monomer and a HEMA unit located on the growing chain close to the radical end in the non-polar solvent, increasing the incorporation rate of the HEMA into the copolymer relative to the bulk system.

While the origin of the enhanced HEMA incorporation in xylene remains a matter of debate, the series of plots in Figure 3 demonstrate that the magnitude of the increased reactivity is accentuated as the solvent fraction is increased from 50 vol% to 90 vol%. However, the change in reactivity is not necessarily proportional to solvent level in general; in DMF and BUOH, a decreased HEMA incorporation is seen as the solvent level is increased from 50% to 80%, but little further change is observed for 90% solvent.

### 3.2. Analysis of Composition-Averaged Propagation Rate Coefficient

**Figure 4:**
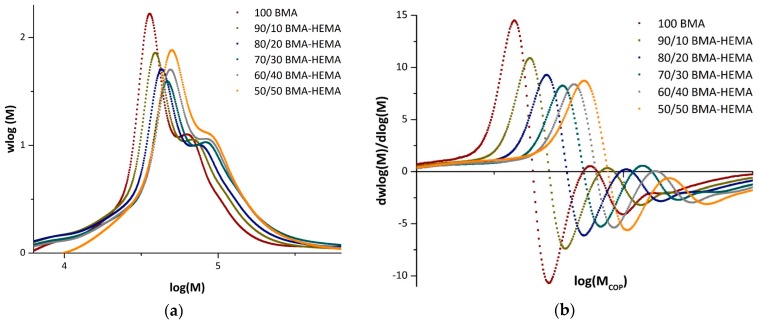
(**a**) Size exclusion chromatography (SEC)-measured molecular weight distributions and (**b**) resultant first derivative curves of BMA/HEMA copolymers generated by pulsed laser polymerization (PLP) of bulk BMA/HEMA mixtures of varying monomer composition. PLP experiments conducted at 80 °C with a pulse repetition rate of 35 Hz using 5 mmol/L 2,2-dimethoxy-2-phenylacetophenone (DMPA).

Copolymer composition provides a means to examine the effect of solvent on relative reactivity. Further insights can be gained by also considering k_p,cop_ values, which measure the solvent influence on absolute (rather than relative) monomer addition rates. Using the IUPAC recommended PLP-SEC method, k_p,cop_ data were collected at two temperatures (50 and 80 °C) and two repetition rates per condition. Figure 4 illustrates the typical PLP structures achieved. With several maxima observed on the corresponding first derivative plots, the first inflection point is taken as the position of MW_1_ used to calculate k_p,cop_ according to Equation (2). To check the validity of the data, the second inflection point is also analyzed to verify that MW_2_ is at about twice the value of the first. The complete set of data are tabulated as Supporting Information (Tables S3–S6).

**Figure 5:**
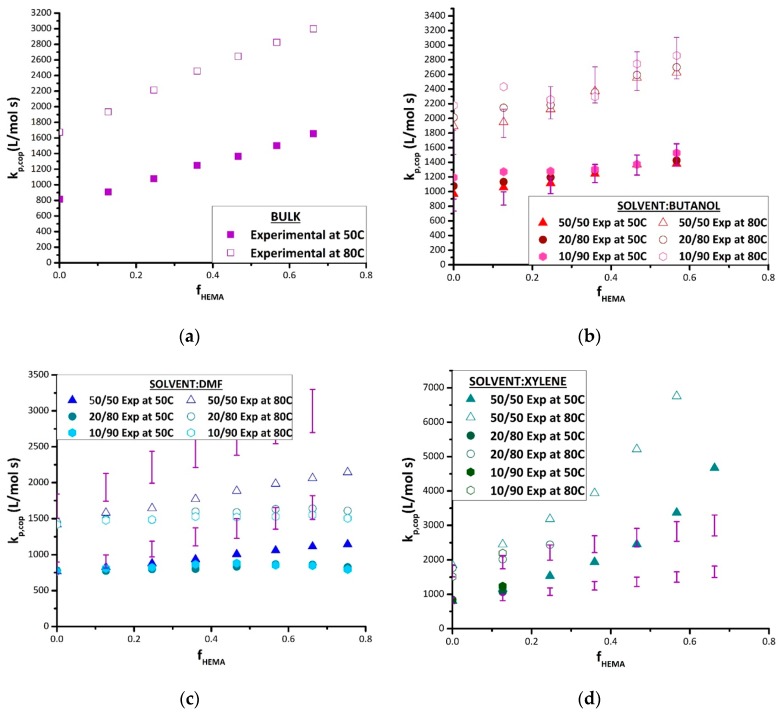
Copolymer k_p,cop_ values plotted against HEMA molar fraction in the BMA/HEMA monomer mixture (f_HEMA_) as measured by PLP/SEC at 50 and 80 °C at different monomer/solvent volume ratios (see legends) in (**a**) bulk, (**b**) *n*-butanol, (**c**) dimethylformamide, and (**d**) xylenes. The results obtained in bulk are included in the plots for solvents with 10% error bars.

The complete set of experimental data is plotted in Figure 5, comparing k_p,cop_ results found in each solvent at both 50 and 80 °C against values obtained for the bulk system, to examine for differences that may be attributed to solvent effects. The values displayed are averaged from the two repetition results per temperature. As previously mentioned, data are not available for HEMA homopolymerization or HEMA-rich copolymers, as the samples were not soluble in THF for SEC analysis. In bulk (Figure 5a), the values of k_p,cop_ increase in a close-to-linear fashion from the BMA homopolymerization value as HEMA is added, as also evidenced by the shift of the distributions (and corresponding inflection points) to higher MWs seen in Figure 4. The same general trend is seen in the various solutions, with the exception of DMF. The plots for the solution data include the bulk values with ±10% error bars, the generally accepted uncertainty in PLP-determined k_p_ values [4,5,6,7,8,9]; deviations outside this range can be attributed to solvent effects rather than experimental error. As H bonds are weaker at higher temperatures, the difference (if any) between the bulk and solvent values may be decreased at 80 °C compared with at 50 °C [12,13].

In BUOH, k_p,cop_ of the BMA/HEMA system is boosted compared with bulk at lower (*f*_HEMA_ < 0.25) HEMA content (Figure 5b). This increase in k_p,cop_ is more pronounced as solvent concentration increases (monomer content lowered from 50 vol% to 10 vol%). In agreement with previously published data [12,15], the increase is dependent on the ratio of BUOH to BMA in the system, and is attributed to an increase in BMA reactivity due to the interactions of its carbonyl group with BUOH. This increased reactivity persists when small amounts of HEMA are added to the system, but disappears completely when HEMA fraction in the monomer mixture is raised to 0.3 (at 50 °C) or 0.2 (at 80 °C). There is no significant difference in k_p,cop_ values between bulk and BUOH solution for higher HEMA contents, although there is some indication that the values in BUOH solution are decreasing to lower than bulk for the higher HEMA fractions of 0.5 and 0.6. These results suggest that BUOH promotes the reactivity of BMA, but may reduce the reactivity of HEMA through its competitive H-bonding, a result also consistent with the reduced HEMA incorporation seen in the copolymer composition data (Figure 3).

As also seen in the copolymer composition results, the relative reactivity of HEMA is greatly reduced in BMA/HEMA copolymerization in DMF relative to the bulk system (Figure 5c). Although k_p,cop_ still increases with increasing HEMA level in 50 vol% DMF, the enhanced reactivity of HEMA completely disappears once DMF becomes the predominant species; for 20 vol% and 10 vol% monomer mixtures in solvent, the k_p,cop_ values are within 10% of the BMA experimental homopropagation value at both 50 and 80 °C. While this disruption of HEMA reactivity has been previously observed in copolymerization with ST [15,30], the complete flattening of the curve in this methacrylate/methacrylate study relative to the bulk system is particularly illuminating, illustrating both the strong influence of HEMA H-bonding on its reactivity in the absence of solvent and its equal reactivity to a “normal” methacrylate such as BMA when that H-bonding is disrupted.

Though an increase in k_p,cop_ values in xylene relative to bulk can be clearly seen (Figure 5d), poor copolymer solubility in the mixtures restricted the experimental range that could be studied at higher HEMA levels. Higher xylene levels (80 vol% and 90 vol%) resulted in turbidity and eventual separation during the low conversion PLP experiment, a result perhaps also exacerbated by the increased incorporation rates of HEMA into the copolymer (see Figure 3). Even at 50 vol% solvent, the reaction cell became cloudy at increased HEMA levels. A second issue limiting the xylene data set was that, even for concentrations for which the solution remained homogeneous, the resultant polymer (containing a high HEMA fraction) was insoluble in THF and thus could not be analyzed by SEC. Previous studies have also found a significant increase in the reactivity of hydroxyfunctional monomers HPMA (hydroxypropyl methacrylate) [13] and HEA [17] when polymerized in xylenes. While all studies relate the behavior to the hydroxyl functional group, the literature has been unable to reach a consensus on the cause of the increased reactivity. It has been hypothesized that a donor–acceptor complex between the growing polymer radical and the aromatic solvent affects reactivity [28,31], and that the non-polar solvent may lead to partial solubility that results in localization of the monomer species and preferential solvation [23,26] or to enhanced H-bonding involving HEMA units in the polymer chain [18]. As discussed for the copolymer composition results, perhaps all of these factors contribute to the behavior observed.

### 3.3. Fitting of Propagation Data

Propagation kinetics in a two monomer system can be represented by the terminal model (Equation (5)) or by the implicit unit model (IPUE) (Equation (6)). While the terminal model assumes that the reactivity of a growing chain is dependent only on the last monomer unit added, the IPUE model assumes that the identity of the unit preceding the radical influences its reactivity (k_p,cop_) but not its selectivity (i.e., copolymer composition) [32]. Thus, the need to use the IPUE to represent k_p,cop_ does not discredit the terminal model representation of composition, and the monomer reactivity ratios estimated from the terminal-model fit of composition data are still employed in the IPUE treatment of k_p,cop_.

(5) kp,cop=r1(f1)2+2f1f2+r2(f2)2r1f1kp,11+r2f2kp,22 ,

(6) kp,cop=r1(f1)2+2f1f2+r2(f2)2r1f1k¯11+r2f2k¯22,

with

(6a) $${\bar{k}}_{11}=\frac{k_{p,111}(r_{1}f_{1}+f_{2})}{r_{1}f_{1}+f_{2}/s_{1}}\;;\;{\bar{k}}_{22}=\frac{k_{p,222}(r_{2}f_{2}+f_{1})}{r_{2}f_{2}+f_{1}/s_{2}}\;;\;s_{1}=\frac{k_{p,211}}{k_{p,111}}\;;\;s_{2}=\frac{k_{p,122}}{k_{p,222}}.$$

The new parameters in the IPUE model, radical reactivity ratios s_1_ and s_2_, capture the influence of the penultimate unit on the reactivity of the radical taking part in the homopropagation of the monomer [32].

For the analysis of k_p,cop_, it is good practice to start by comparing terminal model predictions to the experimental data. While shown not to be valid for many mixed monomer systems (e.g., styrene/methacrylate, styrene/acrylate, acrylate/methacrylate) [14,16,32,33,34,35], the terminal model has provided a reasonable estimate of k_p,cop_ data in the few methacrylate/methacrylate studies that could be found in the literature [32]. However, in order to compare predictions to the data, homopolymerization k_p_ values for each monomer in each solvent are required. This task is not straightforward because, as seen by the data of Figure 5, the homopolymerization endpoints are not only dependent on the monomer/solvent pairing, but also vary with the solvent fraction in the system. Though k_p_ values are experimentally determined for BMA directly, the insolubility of poly(HEMA) in THF made it impossible to measure the corresponding k_p,HEMA_ values. Thus, non-linear parameter estimation was used to estimate the necessary homopolymerization values, assuming that the terminal model (Equation (5)) provides a valid description of the k_p,cop_ datasets and using the reactivity ratios fit from the composition data summarized in Table 2.

**Figure 6:**
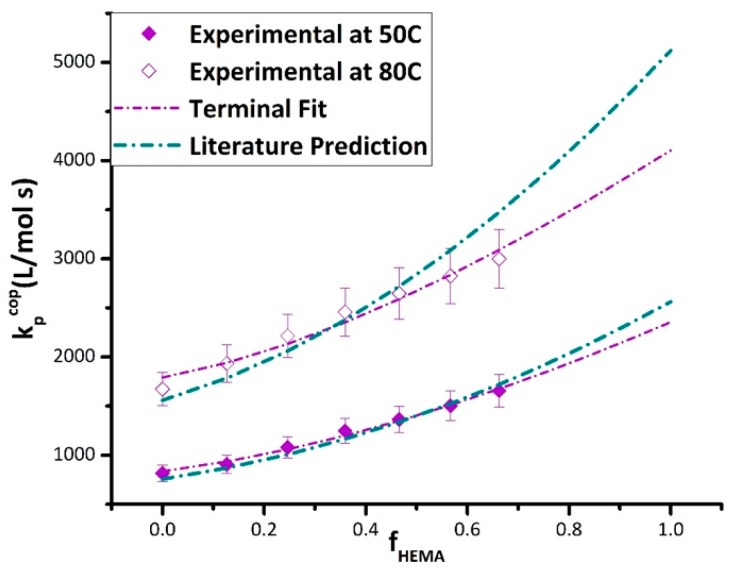
Comparison of k_p,cop_ HEMA/BMA experimental data measured by PLP-SEC experiments at 50 (solid symbols) and 80 °C (open symbols) to terminal model predictions using literature k_p,BMA_ and k_p,HEMA_ values (blue lines), and with best-fit k_p,BMA_ and k_p,HEMA_ values from parameter estimation (purple lines) as summarized in Table 3. Data are shown with 10% error bars.

The fitting methodology was first tested using the bulk k_p,cop_ experimental data plotted in Figure 6, using the bulk reactivity ratios reported in Table 2. The resulting estimates of the BMA (k_p,BMA_) and HEMA (k_p,HEMA_) endpoint values are compared to literature and experimental (available for BMA only) values in Table 3. The agreement with literature values for k_p,BMA_ at both temperatures and with k_p,HEMA_ at 50 °C is within the 10% uncertainty generally associated with the method. However, the discrepancy in the k_p,HEMA_ value estimated at 80 °C with literature [10] is closer to 20%. The corresponding predictions of the terminal model are compared to the experimental bulk k_p,cop_ in Figure 6 over the complete composition range. While the curves generated using the literature k_p_ values go through most of the data points, at 80 °C, the deviation between predicted and experimental becomes greater than the error bars at a higher HEMA content (f_HEMA_ > 0.6). This deviation might be attributed to uncertainty in the HEMA homopropagation k_p_ value, available from a single literature study [10], but could also indicate that the terminal model does not provide a good representation of k_p,cop_ at the higher temperature level. Experimental data measured at a high HEMA content are required to resolve this issue.

**Figure 7:**
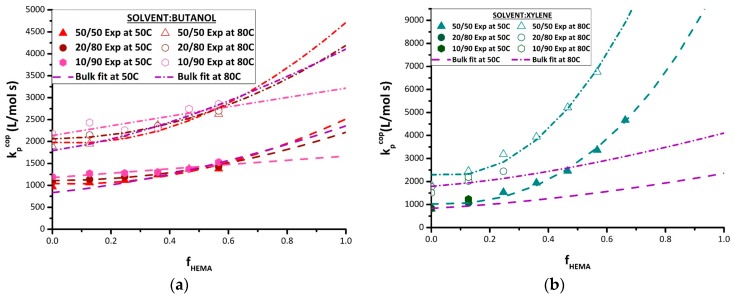
Experimental results (points) for BMA/HEMA k_p,cop_ determined at 50 and 80 °C by PLP-SEC experimentation in (**a**) BUOH and (**b**) xylene at different monomer/solvent volume ratios (see legends). Terminal fits represented by the dotted lines, with best fit values of k_p,BMA_ and k_p,HEMA_ summarized in Table 4. The best-fit lines representing the bulk system are taken from Figure 6.

The strategy developed to fit the terminal model to estimate homopolymer k_p_ values from experimental k_p,cop_ data was then applied to the various sets of solution k_p,cop_ data measured in this study. The best-fit k_p,BMA_ and k_p,HEMA_ values for BUOH and xylene solvents are summarized in Table 4, with the corresponding terminal model curves compared to experimental data in Figure 7. As there were insufficient data for the experiments conducted with 10 vol% and 20 vol% monomer in xylene, the fit was conducted using only the 50 vol% xylene data. While the curve generated by the terminal model fit provides a good representation of the k_p,cop_ data measured in xylene (Figure 7b), it should be noted the best-fit k_p,BMA_ endpoint is higher than the experimental values measured at 80 °C; other studies have also shown that xylene does not influence the value of k_p,BMA_ [15,16]. What is most remarkable is that the value of k_p,HEMA_ required to represent the k_p,cop_ dataset in xylene is greater than the bulk value by a factor of 4–5. While this value is estimated assuming the terminal model, the actual experimental k_p,cop_ values measured at f_HEMA_ = 0.6 already exceed the literature values for bulk HEMA. Although the reason for this behavior is not clear, it cannot be explained by the bootstrap model; the required monomer concentration around the radical would exceed that in a bulk monomer system, a physically impossible situation.

The k_p,cop_ data obtained in BUOH are also well-represented by the terminal model (Figure 7a). Consistent with previous investigations [12,13,15,16] and also matching the experimental data obtained in this study, the estimate of k_p,BMA_ is affected by H-bonding of the monomer with BUOH, with the best-fit value (Table 4) systematically increasing as the monomer fraction is decreased from 50 vol% to 10 vol%. At the other end of the k_p,cop_ curve, the opposite trend is required to fit the data to the terminal model; that is, k_p,HEMA_ is predicted to decrease from the bulk value with increasing BUOH fraction. It is also interesting to note that while higher BUOH content slightly increases the values of k_p,cop_ for BMA-rich mixtures (f_HEMA_ < 0.5), the three curves converge at an f_HEMA_ value of ~0.6, with the increased BUOH content then predicted to decrease the averaged propagation rate coefficient in the system as f_HEMA_ increases further. The ability to analyze PLP-generated HEMA-rich polymer samples is necessary to verify these predictions.

**Figure 8:**
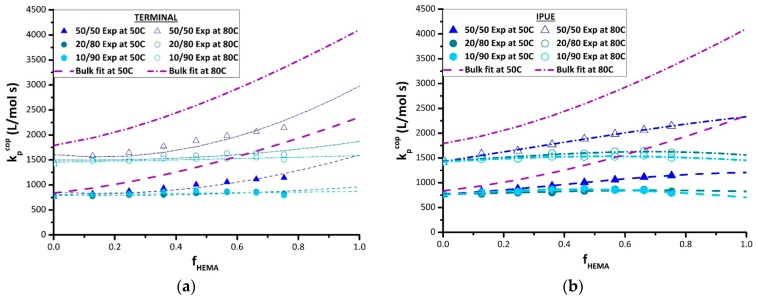
(**a**) Terminal and (**b**) implicit unit (IPUE) model fits (lines) to BMA/HEMA k_p,cop_ data (points) determined at 50 and 80 °C by PLP-SEC experimentation in DMF at different monomer/solvent volume ratios (see legends), with parameter estimates summarized in Table 4 (terminal model) and Table 5 (IPUE).

The results from the terminal model fitting of the BMA/HEMA k_p,cop_ data measured in DMF are also summarized in Table 4, with the resulting curves plotted in Figure 8a. The terminal model captures the disrupting effect of DMF on the reactivity of HEMA, with the k_p,HEMA_ estimates systematically decreasing from the bulk values towards k_p,BMA_ values as the DMF level is increased from 50 vol% to 90 vol%. As the reactivity ratios are close to unity for the 10 vol% and 20 vol% monomer sets, this flattens out the k_p,cop_ curves to match well the experimental k_p,cop_ data. However, the fits to the data obtained with 50 vol% DMF are not as well represented, as seen by comparing the terminal model predictions to the experimental values at 80 °C, and also in the predicted increases in k_p,BMA_ values that are not seen experimentally.

Thus, the penultimate model was also used to fit the k_p,cop_ DMF data, with the best-fit parameters summarized in Table 5 and the fits compared to the experiment in Figure 8b. With more parameters used, the penultimate model provides a better representation of the experimental data. The prediction method, however, showed high uncertainty in the estimation of the reactivity ratios s_1_ and s_2_; as discussed in previous literature [35], precise estimation of these parameters can be quite difficult, especially with an incomplete data set (i.e., no k_p,cop_ values available at high HEMA content). Nonetheless, the penultimate model provides a better fit to the k_p,BMA_ endpoint of the curve. Once again, collecting data at higher HEMA levels is required to determine which curve shape—the concave up prediction of the terminal model fits or the concave down prediction of the penultimate model fits—will match the data over the complete composition range. If the IPUE model proves to be more suitable, it could be theorized that the high polarity and H-bonding cause the penultimate unit to exert steric restrictions [36].

## 4. Conclusions

The PLP technique has been used to investigate the effects of solvents (BUOH, DMF, and xylene) on the low-conversion solution copolymerization propagation kinetics of BMA/HEMA at both 50 and 80 °C. The best-fit monomer reactivity ratios were found to vary not only with solvent type, but also monomer level (50 vol%, 20 vol%, and 10 vol%) in the mixtures. In comparison with the bulk system, there is an increased incorporation of HEMA to the copolymer synthesized in xylene (non-polar solvent) and a decreased incorporation in BUOH and DMF (polar solvents). These effects are attributed to the influence of solvent on H-bonding involving the hydroxyl function present in HEMA. Xylene was found to increase the HEMA reactivity ratio by more than 5-fold in comparison with the bulk value, suggesting a unique behavior in aromatic solvents compared with other relatively non-polar solvents. While DMF resulted in a complete suppression of the increased HEMA incorporation, HEMA was still preferentially incorporated over BMA in BUOH, albeit at a reduced level than seen in bulk.

These relative reactivity tendencies observed from examining the copolymer composition were confirmed in the copolymerization propagation rate (k_p,cop_) kinetics. Propagation rate coefficients in xylene was more than tripled at 50 vol% compared with the bulk system, as HEMA fraction in the HEMA/BMA mixture was increased, with values at higher vol% xylene impossible to attain as a result of polymer solvent immiscibility. The k_p,cop_ behavior observed in BUOH was complex, as BUOH increases k_p,BMA_, but likely reduces k_p,HEMA_; thus the k_p,cop_ values were increased in BUOH compared with bulk for mixtures with low HEMA content (f_HEMA_ < 0.25), but were equal to or slightly lower than bulk values for the remainder of the composition range examined (to f_HEMA_ = 0.6). The disruption of H-bonding in DMF led to the situation where k_p,cop_ values became independent of composition when monomer levels were reduced to 10 vol% and 20 vol% in DMF solution.

In all cases, the observed effects were dependent not only on the solvent choice, but also on the ratio of monomer to solvent; the change in temperature from 50 to 80 °C, on the other hand, did not greatly influence the trends observed. The terminal model was found to sufficiently describe the k_p,cop_ data obtained in bulk and for all solvents, with the IPUE model perhaps more accurate for experimental values in DMF. Unfortunately, the PLP study of k_p,cop_ was limited because of the solubility limits of the polar copolymer (at high HEMA fractions) in THF. Thus, it was not possible to verify the predictions of the terminal model on the effects of solvent concentration on the HEMA homopropagation rate coefficient. Nonetheless, the study provides a significant amount of new data that can be used to test models developed to capture the influence of H-bonding and solvent/monomer interactions on the copolymerization kinetics of hydroxyl-functional monomers. Furthermore, the precise kinetic parameters determined in the study may be applied to simulate HEMA/BMA copolymerization under a range of reaction conditions, as recently demonstrated by applying PLP-measured HEA/BMA kinetic coefficients [16] to the representation of HEA/BMA copolymerization under higher-temperature semi-batch conditions similar to those used in industry [37].

## Figures and Tables

**Table 1:** Parameters required for k_p,cop_ calculation from pulsed laser polymerization (PLP) experiments for butyl methacrylate (BMA)/2-hydroxyethyl methacrylate (HEMA) [14,19], with temperature *T* in °C.

Monomer	Monomer Density (g/mL)	dn/dc (mL/g)	K (dL/g)	a
HEMA	1.092–9.80 × 10^−4^*T*	0.056	2.39 × 10^−4^	0.537
BMA	0.9145–9.64 × 10^−4^*T*	0.080	1.48 × 10^−4^	0.664

**Table 2:** Reactivity ratios determined by terminal model fit to data, with 95% confidence intervals. “System” refers to the vol% of comonomer in the monomer/solvent mixture. BUOH—*n*-butanol; DMF—dimethylformamide; XYL—xylene.

System	r_HEMA_	r_BMA_
Bulk	2.27 ± 0.18	0.66 ± 0.06
50% in BUOH	2.78 ± 0.38	1.10 ± 0.15
20% in BUOH	1.99 ± 0.22	1.00 ± 0.13
10% in BUOH	1.43 ± 0.09	0.69 ± 0.05
50% in DMF	2.39 ± 0.49	1.18 ± 0.28
20% in DMF	1.18 ± 0.12	1.11 ± 0.12
10% in DMF	1.01 ± 0.07	1.00 ± 0.07
50% in XYL	14 ± 5.3	1.97 ± 0.80
20% in XYL	57.6 ± 273	5.41 ± 26
10% in XYL	14.9 ± 7.3	0.75 ± 0.41

**Table 3:** A comparison of BMA and HEMA homopolymerization k_p_ (L/mol s) values in bulk taken from literature [5,10], experimentally determined in this study (BMA only), and estimated (with 95% confidence intervals) according to the terminal model (Equation (5)) with r_HEMA_ = 2.27 and r_BMA_ = 0.66.

Monomer	Literature		Experimental		Fit Values	
	50 °C	80 °C	50 °C	80 °C	50 °C	80 °C
**BMA**	756	1559	816	1673	835 ± 19	1791 ± 63
**HEMA**	2563	5123			2355 ± 56	4105 ± 142

**Table 4:** Homopropagation k_p_ (L/mol s) values (with 95% confidence intervals) estimated by fitting of the terminal model to BMA/HEMA k_p,cop_ data obtained in xylene (XYL), butanol (BUOH), and DMF solution at 50 and 80 °C, with reactivity ratios for each system taken from Table 2. Percentage values represent the vol% of comonomer mixture in the solvent.

System	k_p,HEMA_		k_p,BMA_	
	50 °C	80 °C	50 °C	80 °C
Bulk	2355 ± 56	4105 ± 142	835 ± 19	1791 ± 63
50% in XYL	11,152 ± 1066	19,463 ± 2331	1025 ± 81	2297 ± 191
50% in BUOH	2513 ± 214	4712 ± 53	1039 ± 340	1980 ± 86
20% in BUOH	2211 ± 87	4189 ± 24	1109 ± 141	2060 ± 38
10% in BUOH	1667 ± 79	3216 ± 39	1177 ± 284	2138 ± 129
50% in DMF	1599 ± 86	2978 ± 170	853 ± 42	1606 ± 85
20% in DMF	963 ± 32	1870 ± 63	794 ± 19	1499 ± 35
10% in DMF	872 ± 39	1585 ± 29	808 ± 25	1455 ± 19

**Table 5:** Homopropagation k_p_ values (L/mol s) and radical reactivity ratios (with 95% confidence intervals) estimated by fitting of the IPUE model to BMA/HEMA k_p,cop_ data measured in DMF solution at 50 and 80 °C, with monomer reactivity ratios for each system taken from Table 2. Percentage values represent the vol% of comonomer mixture in DMF.

System	50% in DMF		20% in DMF		10% in DMF	
	50 °C	80 °C	50 °C	80 °C	50 °C	80 °C
k_p,HEMA_	1261 ± 41	2332 ± 88	837 ± 131	1584 ± 182	704 ± 73	1454 ± 113
k_p,BMA_	763 ± 8.6	1426 ± 19	766 ± 212	1437 ± 31	759 ± 16	1421 ± 17
s_HEMA_	2.37 ± 1.74	3.2 ± 3.7	0.9 ± 2.5	0.89 ± 1.96	1.6 ± 112	0.88 ± 26
s_BMA_	2.15 ± 1.44	1.99 ± 1.42	2.3 ± 18	2.5 ± 16	1.4 ± 87	1.6 ± 87

## Supplementary Materials

The data tables discussed in the text are available at https://www.mdpi.com/2073-4360/11/3/487/s1. **Table S1**: Molar fraction of HEMA (F_HEMA_) in BMA/HEMA copolymers produced of low conversion by PLP in bulk and in n-butanol (BUOH) and dimethyl formamide (DMF) (vol% monomer) as a function of HEMA molar fraction (f_HEMA_) in the monomer mixture, **Table S2**: Molar fraction of HEMA (F_HEMA_) in BMA/HEMA copolymers produced at low conversion by PLP in xylene (XYL) as a function of HEMA molar fraction (f_HEMA_) in the monomer mixture, **Table S3**: k_p,cop_ values obtained from PLP-SEC experiments of BMA/HEMA in bulk with 5 mmol/L DMPA photoinitiator as a function of mol fraction HEMA in the monomer mixture (f_HEMA_), temperature and laser pulse repetition rate (f_HEMA_). L_1_/L_2_ is the ratio of the first two inflection points on the PLP-produced MMD, **Table S4**: k_p,cop_ values obtained from PLP-SEC experiments of BMA/HEMA in n-butanol with 5 mmol/L DMPA photoinitiator as a function of mol fraction HEMA in the monomer mixture (f_HEMA_), temperature and laser pulse repetition rate (f_HEMA_). L_1_/L_2_ is the ratio of the first two inflection points on the PLP-produced MMD, **Table S5**: k_p,cop_ values obtained from PLP-SEC experiments of BMA/HEMA in dimethylformamide with 5 mmol/L DMPA photoinitiator as a function of mol fraction HEMA in the monomer mixture (f_HEMA_), temperature and laser pulse repetition rate. L_1_/L_2_ is the ratio of the first two inflection points on the PLP-produced MMD, **Table S6**: k_p,cop_ values obtained from PLP-SEC experiments of BMA/HEMA in xylene with 5 mmol/L DMPA photoinitiator as a function of mol fraction HEMA in the monomer mixture (f_HEMA_), temperature, laser pulse repetition rate and vol % monomer. L_1_/L_2_ is the ratio of the first two inflection points on the PLP-produced MMD.
