# Supplementary material for: Solvent Effects on Radical Copolymerization Kinetics of 2-Hydroxyethyl Methacrylate and Butyl Methacrylate
Source: Polymers (Basel). 2019 Mar 13;11(3):487. doi: 10.3390/polym11030487 (PMC6474149; doi:10.3390/polym11030487)
Supplement: Supplementary file 1 [file polymers-11-00487-s001.pdf]

Solvent Effects on Radical Copolymerization Kinetics of 2-Hydroxyethyl Methacrylate and Butyl Methacrylate

Loretta A. Idowu and Robin A. Hutchinson *

Department of Chemical Engineering, Queen's University, 19 Division St., Kingston ON K7L 3N6 Canada

* Correspondence: robin.hutchinson@queensu.ca

Supporting Information

Table S1: Molar fraction of HEMA (F_{HEMA}) in BMA/HEMA copolymers produced of low conversion by PLP in bulk and in *n*-butanol (BUOH) and dimethyl formamide (DMF) (vol% monomer) as a function of HEMA molar fraction (f_{HEMA}) in the monomer mixture.

	F_{HEMA}	F_{HEMA}	F_{HEMA}	F_{HEMA}	F_{HEMA}	F_{HEMA}	F_{HEMA}
f_{HEMA}	BULK	50% mon in BUOH	20% mon in BUOH	10% mon in BUOH	50% mon in DMF	20% mon in DMF	10% mon in DMF
**0.13**	0.22	0.14	-	0.17	-	0.12	0.13
**0.25**	0.35	0.32	0.30	0.31	0.29	0.26	0.25
**0.36**	0.50	0.45	0.44	0.45	0.46	0.37	0.38
**0.47**	0.63	0.59	0.55	0.59	0.53	0.43	0.45
**0.57**	0.72	0.74	0.67	0.66	0.64	0.59	0.56
**0.66**	0.81	-	0.75	0.75	0.81	0.68	0.66
**0.75**	0.88	-	0.87	0.80	0.85	0.77	0.75
**0.84**	-	-	-	0.86	0.95	0.86	0.85
**0.92**	-	-	-	0.94	-	0.96	0.95

Table S2: Molar fraction of HEMA (F_{HEMA}) in BMA/HEMA copolymers produced at low conversion by PLP in xylene (XYL) as a function of HEMA molar fraction (f_{HEMA}) in the monomer mixture.

	F_{HEMA}	F_{HEMA}	F_{HEMA}
f_{HEMA}	50% mon in XYL	20% mon in XYL	10% mon in XYL
**0.13**	0.21	0.27	0.32
**0.25**	0.43	0.42	0.69
**0.36**	0.63	0.85	0.75
**0.47**	0.81	0.88	0.89
**0.57**	0.91	-	-
**0.66**	0.97	-	-
**0.75**	-	-	-

Table S3: k_p^{cop} values obtained from PLP-SEC experiments of BMA/HEMA in bulk with 5 mmol/L DMPA photoinitiator as a function of mol fraction HEMA in the monomer mixture (f_{HEMA}), temperature and laser pulse repetition rate (ν). L_1/L_2 is the ratio of the first two inflection points on the PLP-produced MMD.

f_{HEMA}	T (°C)	ν (Hz)	k_p (L/mol s)	L_1/L_2	f_{HEMA}	T (°C)	ν (Hz)	k_p (L/mol s)	L_1/L_2
**0**	50	20	764	0.51	**0**	80	35	1626	0.51
**0.13**	50	20	863	0.49	**0.13**	80	35	1793	0.50
**0.25**	50	20	1043	0.50	**0.25**	80	35	2069	0.50
**0.36**	50	20	1150	0.49	**0.36**	80	35	2333	0.49
**0.47**	50	20	1267	0.49	**0.47**	80	35	2511	0.49
**0**	50	40	840	0.52	**0.57**	80	35	2702	0.48
**0.13**	50	40	927	0.52	**0**	80	70	1668	0.52
**0.25**	50	40	1070	0.52	**0.13**	80	70	1970	0.52
**0.36**	50	40	1264	0.52	**0.25**	80	70	2274	0.52
**0.47**	50	40	1393	0.51	**0.36**	80	70	2564	0.52
**0.57**	50	40	1432	0.51	**0.47**	80	70	2635	0.51
**0**	50	80	842	0.49	**0.57**	80	70	2836	0.52
**0.13**	50	80	929	0.49	**0.66**	80	70	2913	0.51
**0.25**	50	80	1123	0.51	**0**	80	100	1726	0.52
**0.36**	50	80	1327	0.54	**0.13**	80	100	2039	0.54
**0.47**	50	80	1429	0.52	**0.25**	80	100	2300	0.52
**0.57**	50	80	1574	0.52	**0.36**	80	100	2476	0.52
**0.66**	50	80	1655	0.54	**0.47**	80	100	2791	0.52
					**0.57**	80	100	2935	0.51
					**0.66**	80	100	3085	0.52

Table S4: k_p^{cop} values obtained from PLP-SEC experiments of BMA/HEMA in *n*-butanol with 5 mmol/L DMPA photoinitiator as a function of mol fraction HEMA in the monomer mixture (f_{HEMA}), temperature and laser pulse repetition rate (ν). L_1/L_2 is the ratio of the first two inflection points on the PLP-produced MMD.

		50 vol% Monomer in BUOH			20 vol% Monomer in BUOH			10 vol% Monomer in BUOH		
f_{HEMA}	T (°C)	ν (Hz)	k_p (L/mol s)	L_1/L_2	ν (Hz)	k_p (L/mol s)	L_1/L_2	ν (Hz)	k_p (L/mol s)	L_1/L_2
**0**	50	20	968	0.52	4	1037	0.50	8	1198	0.51
**0.13**	50	20	1068	0.54	4	1093	0.49	8	1292	0.51
**0.25**	50	20	1075	0.52	4	1152	0.49	8	1300	0.52
**0.36**	50	20	1213	0.52	4	1241	0.48	8	1278	0.52
**0.47**	50	20	1306	0.52	4	1337	0.48	8	1285	0.50
**0.57**	50	20	1313	0.51	4	1408	0.47	8	1552	0.52
**0**	50	40	971	0.50	8	1114	0.52	12	1187	0.48
**0.13**	50	40	1047	0.50	8	1174	0.52	12	1251	0.48
**0.25**	50	40	1154	0.51	8	1237	0.51	12	1259	0.47
**0.36**	50	40	1273	0.51	8	1333	0.52	12	1326	0.47
**0.47**	50	40	1436	0.54	8	1404	0.51	12	1462	0.47
**0.57**	50	40	1443	0.52	8	1444	0.51	12	1503	0.48
**0**	80	35	1891	0.54	7	1942	0.49	10	2213	0.52
**0.13**	80	35	1992	0.52	7	2094	0.49	10	2387	0.51
**0.25**	80	35	2050	0.52	7	2107	0.49	10	2294	0.52
**0.36**	80	35	2260	0.51	7	2271	0.49	10	2307	0.52
**0.47**	80	35	2434	0.52	7	2561	0.48	10	2726	0.52
**0.57**	80	35	2502	0.51	7	2634	0.48	10	2740	0.51
**0**	80	70	1895	0.50	14	2086	0.52	15	2144	0.49
**0.13**	80	70	1907	0.48	14	2198	0.52	15	2477	0.51
**0.25**	80	70	2202	0.51	14	2263	0.51	15	2221	0.49
**0.36**	80	70	2484	0.52	14	2439	0.52	15	2286	0.50
**0.47**	80	70	2675	0.54	14	2627	0.50	15	2764	0.51
**0.57**	80	70	2750	0.54	14	2765	0.51	15	2977	0.51

Table S5: k_p^{cop} values obtained from PLP-SEC experiments of BMA/HEMA in dimethylformamide with 5 mmol/L DMPA photoinitiator as a function of mol fraction HEMA in the monomer mixture (f_{HEMA}), temperature and laser pulse repetition rate (ν). L_1/L_2 is the ratio of the first two inflection points on the PLP-produced MMD.

		50 vol% Monomer in DMF			20 vol% Monomer in DMF			10 vol% Monomer in DMF		
f_{HEMA}	T (°C)	ν (Hz)	k_p (L/mol s)	L_1/L_2	ν (Hz)	k_p (L/mol s)	L_1/L_2	ν (Hz)	k_p (L/mol s)	L_1/L_2
**0**	50	40	712	0.49	4	734	0.50	4	749	0.50
**0.13**	50	40	768	0.49	4	738	0.49	4	789	0.50
**0.25**	50	40	828	0.49	4	760	0.49	4	812	0.50
**0.36**	50	40	892	0.49	4	782	0.49	4	855	0.50
**0.47**	50	40	983	0.50	4	823	0.49	4	880	0.48
**0.57**	50	40	1034	0.50	4	866	0.48	4	864	0.49
**0.66**	50	40	1088	0.51	4	831	0.48	4	868	0.49
**0.75**	50	40	1117	0.51	4	797	0.50	4	795	0.47
**0**	50	80	820	0.54	8	825	0.52	8	786	0.49
**0.13**	50	80	884	0.52	8	812	0.49	8	828	0.49
**0.25**	50	80	931	0.52	8	835	0.50	8	833	0.49
**0.36**	50	80	980	0.51	8	821	0.50	8	877	0.49
**0.47**	50	80	1031	0.51	8	844	0.50	8	882	0.49
**0.57**	50	80	1085	0.50	8	868	0.50	8	846	0.47
**0.66**	50	80	1141	0.49	8	893	0.50	8	831	0.48
**0.75**	50	80	1173	0.49	8	856	0.50	8	797	0.47

Table S5 continued on following page

Table S5 (continued from previous page)

		50 vol% Monomer in DMF			20 vol% Monomer in DMF			10 vol% Monomer in DMF		
f_{HEMA}	T (°C)	ν (Hz)	k_p (L/mol s)	L_1/L_2	ν (Hz)	k_p (L/mol s)	L_1/L_2	ν (Hz)	k_p (L/mol s)	L_1/L_2
**0**	80	70	1376	0.49	7	1447	0.49	10	1424	0.50
**0.13**	80	70	1553	0.50	7	1490	0.49	10	1501	0.51
**0.25**	80	70	1636	0.50	7	1464	0.49	10	1509	0.51
**0.36**	80	70	1762	0.50	7	1577	0.49	10	1553	0.51
**0.47**	80	70	1897	0.51	7	1586	0.50	10	1561	0.51
**0.57**	80	70	2042	0.51	7	1593	0.49	10	1569	0.51
**0.66**	80	70	2098	0.51	7	1600	0.49	10	1576	0.50
**0.75**	80	70	2205	0.52	7	1607	0.49	10	1511	0.50
**0**	80	100	1458	0.49	14	1450	0.50	15	1411	0.49
**0.13**	80	100	1608	0.49	14	1493	0.50	15	1453	0.47
**0.25**	80	100	1654	0.49	14	1502	0.50	15	1462	0.47
**0.36**	80	100	1782	0.49	14	1618	0.50	15	1504	0.49
**0.47**	80	100	1875	0.48	14	1589	0.50	15	1478	0.48
**0.57**	80	100	1927	0.48	14	1672	0.50	15	1485	0.48
**0.66**	80	100	2026	0.49	14	1680	0.50	15	1526	0.48
**0.75**	80	100	2081	0.49	14	1610	0.50	15	1498	0.48

Table S6: k_p^{cop} values obtained from PLP-SEC experiments of BMA/HEMA in xylene with 5 mmol/L DMPA photoinitiator as a function of mol fraction HEMA in the monomer mixture (f_{HEMA}), temperature, laser pulse repetition rate (ν) and vol % monomer. L_1/L_2 is the ratio of the first two inflection points on the PLP-produced MMD.

f_{HEMA}	T (°C)	ν (Hz)	Vol% Mon	k_p (L/mol s)	L_1/L_2	f_{HEMA}	T (°C)	ν (Hz)	Vol% Mon	k_p (L/mol s)	L_1/L_2
**0.00**	50	20	50	822	0.52	**0.00**	80	35	50	809	0.52
**0.13**	50	20	50	1115	0.51	**0.13**	80	35	50	1156	0.50
**0.25**	50	20	50	1513	0.49	**0.25**	80	35	50	1533	0.51
**0.36**	50	20	50	1960	0.55	**0.36**	80	35	50	1940	0.66
**0.47**	50	20	50	2369	0.48	**0.47**	80	35	50	2457	0.46
**0.57**	50	20	50	3287	0.47	**0.57**	80	35	50	3368	0.44
**0.66**	50	20	50	4664	0.44	**0.00**	80	70	50	1875	0.50
**0.00**	50	40	50	796	0.49	**0.13**	80	70	50	2588	0.51
**0.13**	50	40	50	1198	0.51	**0.25**	80	70	50	3297	0.51
**0.25**	50	40	50	1552	0.54	**0.36**	80	70	50	4078	0.48
**0.36**	50	40	50	1920	0.50	**0.47**	80	70	50	5403	0.48
**0.47**	50	40	50	2545	0.49	**0.57**	80	70	50	7156	0.48
**0.57**	50	40	50	3450	0.49	**0.00**	80	7	20	1725	0.52
**0.66**	50	40	50	4675	0.46	**0.13**	80	7	20	1948	0.50
**0.00**	50	4	20	801	0.52	**0.25**	80	7	20	2356	0.49
**0.13**	50	4	20	1039	0.52	**0.00**	80	14	20	1811	0.55
**0.00**	50	8	20	881	0.55	**0.13**	80	14	20	2092	0.52
**0.13**	50	8	20	1090	0.51	**0.25**	80	14	20	2531	0.54
**0.00**	50	8	10	805	0.50	**0.00**	80	10	10	1468	0.50
**0.13**	50	8	10	1255	0.51	**0.13**	80	10	10	2236	0.54
**0.00**	50	12	10	855	0.52	**0.00**	80	15	10	1559	0.51
**0.13**	50	12	10	1215	0.47	**0.13**	80	15	10	2152	0.49
